# Supplementary material for: Transcriptome Analysis of Glycerin Regulating Reuterin Production of Lactobacillus reuteri
Source: Microorganisms. 2023 Aug 4;11(8):2007. doi: 10.3390/microorganisms11082007 (PMC10459645; doi:10.3390/microorganisms11082007)
Supplement: Supplementary file 1 [file microorganisms-11-02007-s001.zip › microorganisms-2496760-supplementary.pdf]

Supplementary materials

Formula of protein purification buffer

Wash buffer: 20 mM Tris-HCl buffer, 100 mM NaCl, 10% glycerol (v/v), 7 mM 2-ME, and 20 mM imidazole. pH 7.0.
Elution buffer: 20 mM Tris-HCl buffer, 100 mM NaCl, 10% glycerol (v/v), 7 mM 2-ME, and 250 mM imidazole. pH 7.0.
Saving buffer: 20 mM Tris-HCl buffer, 100 mM NaCl, 10% glycerol (v/v), and 7 mM 2-ME. pH 7.0.
The prepared solution is degassed by ultrasonic after suction filtration.

Specific steps of protein purification

(1) The crude enzyme solution was filtered using filter membrane with 0.22 μm of aperture, and added to Ni-column.
(2) After the supernatant is flowed clean, ten times the volume of wash buffer was added to the column to washout the mixed protein.
(3) Add three times the column volume of elution buffer to elute the target protein, and start to collect the target protein in separate tubes, adjust the concentration of imidazole from 50 to 250 mM, and collect the protein eluate from low to high concentrations in turn.
(4) Using 15% SDS-polyacrylamide gel (SDS-PAGE) to detect the purity of protein in the collected eluate, and the eluate containing only a single target band was collected and mixed. At the same time, the purified protein was compared to that of wild type fermentation broth of *Lactobacillus reuteri* LR301.
(5) Put the mixed solution in a dialysis bag, and put the dialysis bag in a saving buffer at 4°C for dialysis to replace the *GDHt* storage system. The dialysis buffer was changed every 5 hours and dialyzed three times continuously to obtain pure *GDHt* solution.

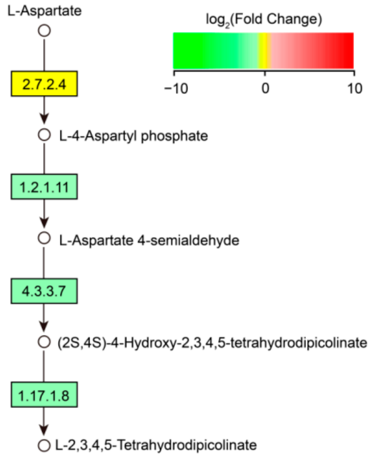

Figure S1. Changed genes participating in monobactam biosynthesis in *Lactobacillus reuteri* treated by 600 mM glycerol. Green indicates that the transcription levels of genes are significantly down-regulated ($p < 0.05$). Yellow indicates that the transcription levels of genes are no significantly changed ($p \geq 0.05$).

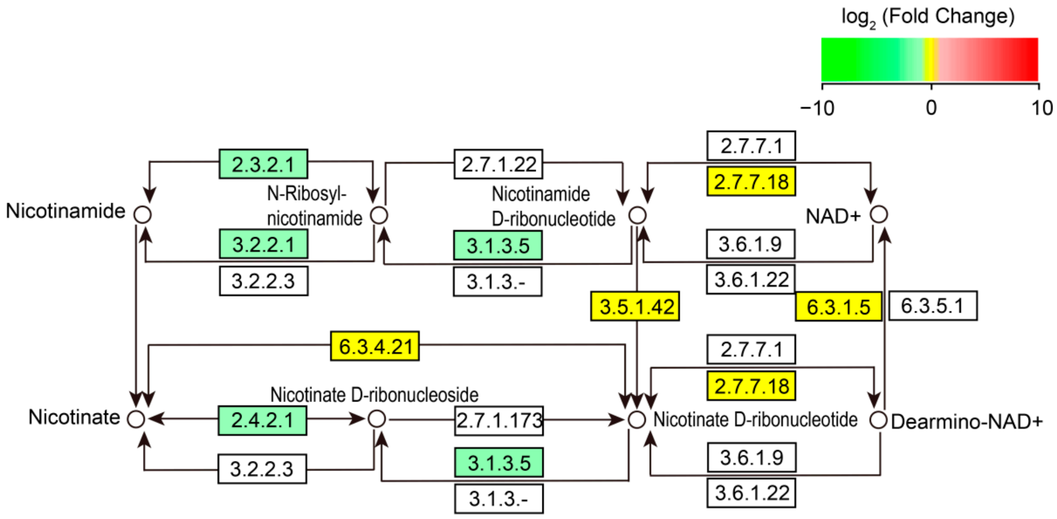

Figure S2. Changed genes participating in nicotinate and nicotinamide metabolism in *Lactobacillus reuteri* treated by 600 mM glycerol. Green indicates that the transcription levels of genes are significantly down-regulated ($p < 0.05$). Yellow indicates that the transcription levels of genes are no significantly changed ($p \geq 0.05$).

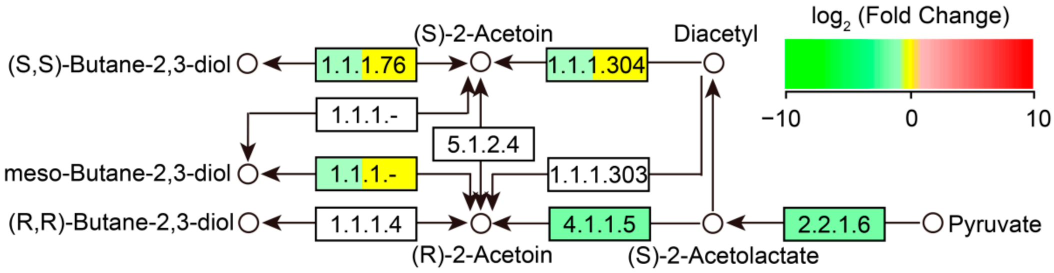

Figure S3. Changed genes participating in butanoate metabolism in *Lactobacillus reuteri* treated by 600 mM glycerol. Green indicates that the transcription levels of genes are significantly down-regulated ($p < 0.05$). Yellow indicates that the transcription levels of genes are no significantly changed ($p \geq 0.05$).

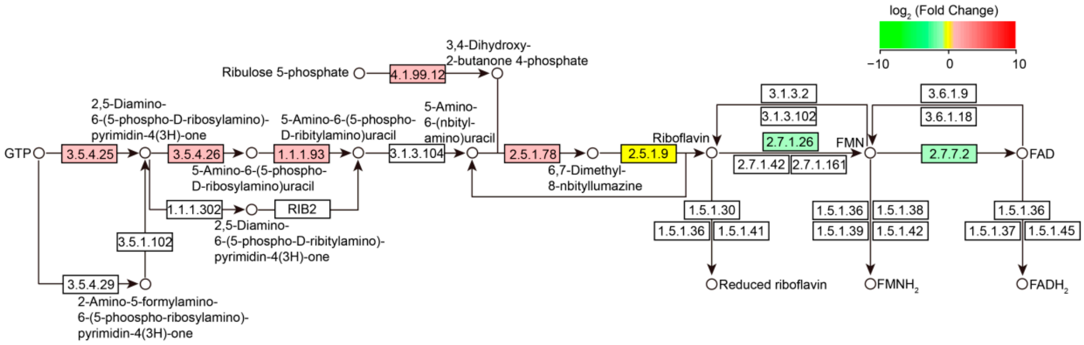

Figure S4. Changed genes participating in riboflavin metabolism in *Lactobacillus reuteri* treated by 600 mM glycerol. Red indicates that the transcription levels of genes are significantly up-regulated ($p < 0.05$). Green indicates that the transcription levels of genes are significantly down-regulated ($p < 0.05$). Yellow indicates that the transcription levels of genes are no significantly changed ($p \geq 0.05$).

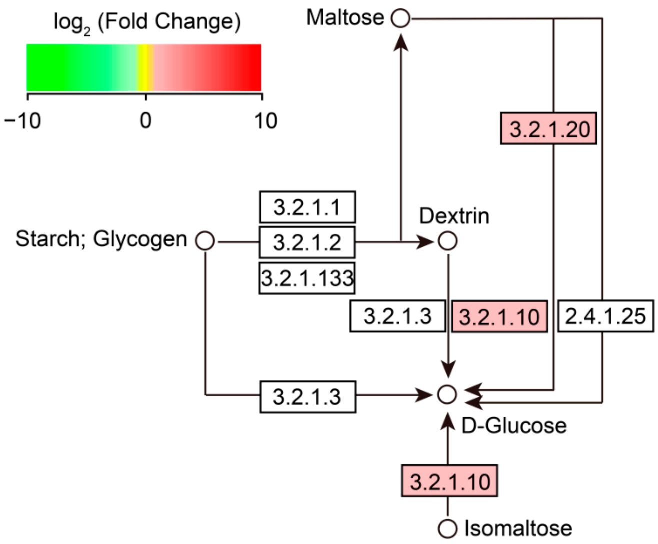

Figure S5. Changed genes participating in starch and sucrose metabolism in *Lactobacillus reuteri* treated by 600 mM glycerol. Red indicates that the transcription levels are significantly up-regulated ($p < 0.05$).
